# Regional Anesthesia for Painful Injuries after Disasters (RAPID): study protocol for a randomized controlled trial

**DOI:** 10.1186/s13063-016-1671-z

**Published:** 2016-11-14

**Authors:** Adam C. Levine, Carrie Teicher, Adam R. Aluisio, Tess Wiskel, Pola Valles, Miguel Trelles, Justin Glavis-Bloom, Rebecca F. Grais

**Affiliations:** 1 Warren Alpert School of Medicine, Brown University, 55 Claverick Street, Room 274, Providence, RI 02903 USA; 2 Epicentre, Paris, France; 3 Médecins Sans Frontières Belgium, Brussels, Belgium

**Keywords:** Natural disaster, Earthquake, Regional anesthesia, Pain management, Humanitarian response, Randomized controlled trial

## Abstract

**Background:**

Lower extremity trauma during earthquakes accounts for the largest burden of disaster-related injuries. Insufficient pain management is common in resource-limited disaster settings, and regional anesthesia (RA) may reduce pain in injured patients beyond current standards of care. To date, no controlled trials have been conducted to evaluate the use of RA for pain management in a disaster setting.

**Methods/design:**

The Regional Anesthesia for Painful Injuries after Disasters (RAPID) study aims to evaluate whether regional anesthesia (RA), either with or without ultrasound (US) guidance, can reduce pain from earthquake-related lower limb injuries in a disaster setting. The proposed study is a blinded, randomized controlled equivalence trial among earthquake victims with serious lower extremity injuries in a resource-limited setting. After obtaining informed consent, study participants will be randomized in a 1:1:1 allocation to either: standard care (parenteral morphine at 0.1 mg/kg); standard care plus a landmark-guided fascia iliaca compartment block (FICB); or standard care plus an US-guided femoral nerve block. General practice humanitarian response providers who have undergone a focused training in RA will perform nerve blocks with 20 ml 0.5 % levobupivacaine. US sham activities will be used in the standard care and FICB arms and a normal saline injection will be given to the control group to blind both participants and nonresearch team providers. The primary outcome measure will be the summed pain intensity difference calculated using a standard 11-point Numerical Rating Scale reported by patients over 24 h of follow-up. Secondary outcome measures will include overall analgesic requirements, adverse events, and participant satisfaction.

**Discussion:**

Given the high burden of lower extremity injuries in the aftermath of earthquakes and the currently limited treatment options, research into adjuvant interventions for pain management of these injuries is necessary. While anecdotal reports on the use of RA for patients injured during earthquakes exist, no controlled studies have been undertaken. If demonstrated to be effective in a disaster setting, RA has the potential to significantly assist in reducing both acute suffering and long-term complications for survivors of earthquake trauma.

**Trial registration:**

ClinicalTrials.gov (NCT02698228), registered on 16 February 2016.

## Background

Between 1994 and 2013, approximately 7000 natural disasters were reported globally, affecting more than 200 million people and accounting for over one million deaths. Earthquakes, and their resultant morbidity and mortality, have increased over time due to population growth and increasing urbanization. Given these trends, earthquakes now account for the largest burden of injury among all types of geophysical disasters [1, 2].

Epidemiologic studies from multiple settings consistently show that trauma sustained during earthquakes predominantly injures the extremities, with the lower limbs most commonly affected [3–6]. The high frequency of lower limb trauma during earthquakes makes interventions directed at the management of these injuries critical [7].

Inadequate pain management due to oligoanalgesia is common in nondisaster acute care treatment [8–10]. In the emergency response phase to a natural disaster, where resources are often constrained, oligoanalgesia is even more common [11–13]. Standard pain management in disaster settings for severe lower limb injuries includes intramuscular or intravenous injections of narcotic pain medications, with morphine being the recommended agent [14, 15]. However, narcotic medications may be unavailable in low-income settings, and patients with trauma may have clinical instability making narcotic medications less safe to use; both factors can be barriers to appropriate pain treatment for earthquake victims [11, 16]. Subsequently, insufficient pain management can potentiate both short-term and long-term sequelae, including immunosuppression, thrombotic complications, post-traumatic stress disorders, and chronic pain syndromes, all of which may further increase the morbidity associated with index injuries [12, 17].

Regional anesthesia (RA) has the potential to address the suboptimal pain management that often occurs after earthquakes. RA involves injecting local anesthetic medications around a nerve in order to block sensation to a specific anatomic region [18]. Prior studies have demonstrated that RA to achieve femoral nerve blockade is a rapid and safe method for pain management of lower extremity trauma [19–23]. Hospital-based studies have found RA to be more effective at reducing pain as compared to narcotics alone in patients with limb injuries. However, nearly all studies on RA have been conducted in high-resource settings, using specially trained proceduralists, and have enrolled only patients with simple hip or femoral fractures [24–26]. This population is not representative of earthquake victims, who more frequently have complex injuries including multiple, open fractures and crush mechanisms, thereby limiting the generalizability of these RA findings to disaster response settings.

RA can be achieved using either anatomic landmark or ultrasound (US) guidance. Both the anatomically guided fascia iliaca compartment block (FICB) and the US-guided femoral nerve block (FNB) primarily target the femoral nerve, and with injection of an anesthetic agent can provide analgesia for much of the lower extremity [18]. A review article concluded that US-guided nerve blocks have the potential to greatly improve pain control in disaster settings [17]. However, the published literature on the use of US-guided RA in disasters is limited, and no controlled trials have evaluated the effectiveness of RA in a disaster context or resource-limited setting. A few studies have suggested that local anesthetic injection using US may reduce complication rates compared to anatomic landmark-guided injection [27]. However, a systematic review found no evidence that US-guided RA was more effective than the use of landmark-guided injections [27]. Furthermore, the anatomic method does not require investment in costly US equipment, which may be important in a resource-limited or disaster setting.

There are anecdotal reports of the use of both anatomic landmark- and US-guided RA for the treatment of earthquake-related injuries, suggesting the feasibility of these modalities in a disaster setting. However, there have been no randomized controlled trials (RCTs) conducted to evaluate the effectiveness, safety, or acceptability of RA in the aftermath of a major earthquake [13, 28, 29]. Given the burden of disease, consistent patterns of injuries, and inadequate pain treatment documented in earthquake settings, interventions to address this substantial global health burden are needed. A rigorous assessment of the use of RA techniques to address pain management in disaster settings would be useful to inform treatment protocols. To address this current deficit, the Regional Anesthesia for Painful Injuries after Disasters (RAPID) study aims to enroll patients in the aftermath of a major earthquake to determine whether regional anesthesia, either with or without ultrasound guidance, can reduce suffering from lower limb injuries, the most common earthquake-related injury, as compared to current standard of care for pain control in these settings.

## Methods

### Design

**Fig. 1:**
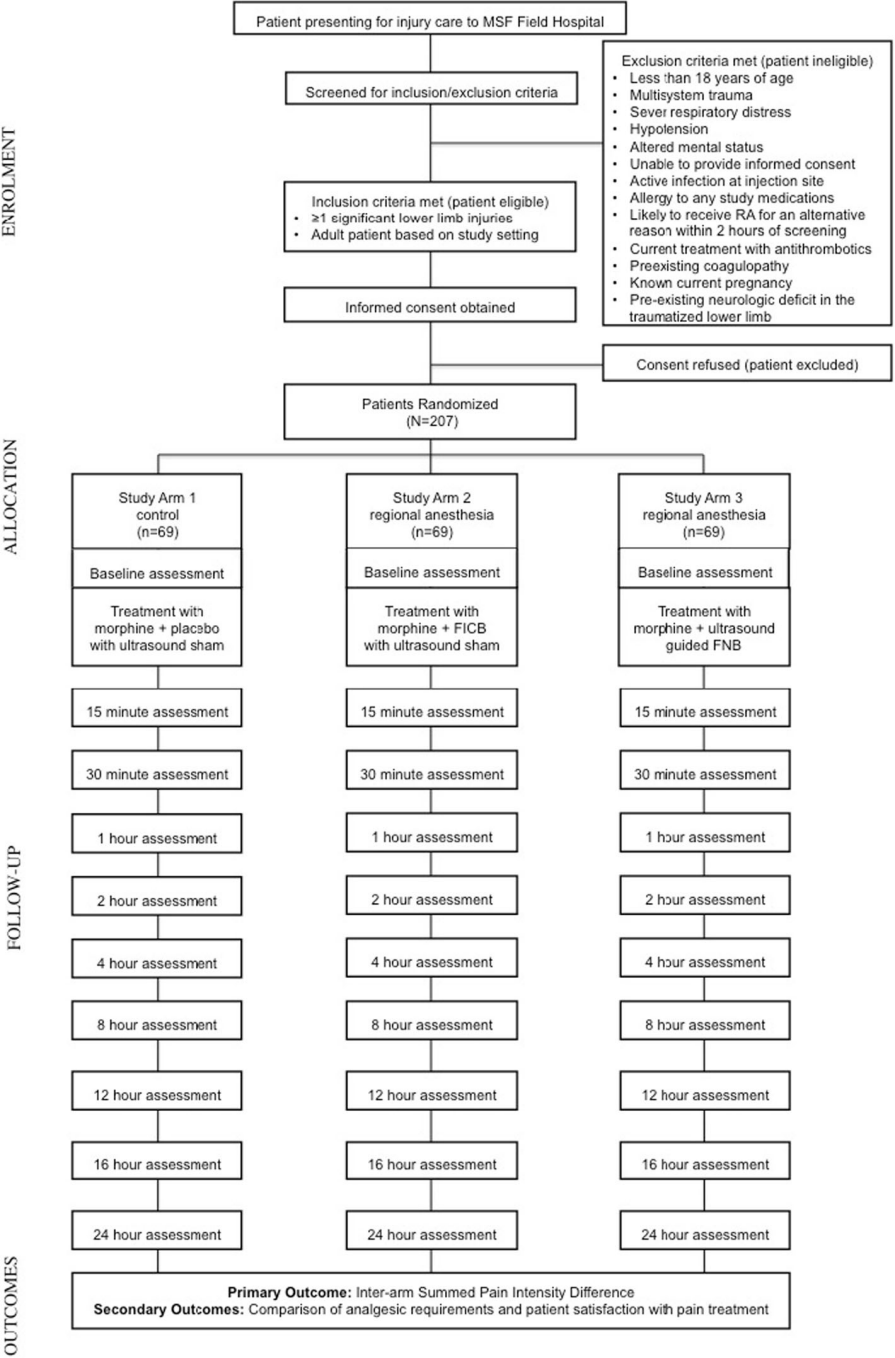
Study flow diagram. Abbreviations: *MSF* Médecins Sans Frontières

The RAPID study will be conducted in the aftermath of a major earthquake to which a Médecins Sans Frontières (MSF) field hospital is deployed at the request of the local Ministry of Health (MoH). The study design is a blinded RCT using an equivalence design. After obtaining informed consent, earthquake victims with serious lower extremity injuries will be randomized to one of three study arms. Eligible participants will be randomized in a 1:1:1 allocation ratio to either: standard care (defined below); standard care plus an anatomic landmark-guided FICB; or standard care plus an US-guided FNB. The structure of the study is illustrated in Fig. 1.

### Aims

The goal of the RAPID study is to transform the way that serious injuries are managed after earthquakes and other disasters by introducing a cost-effective and anatomically appropriate method for pain control. The trial will enroll patients in the aftermath of a major earthquake to determine whether RA, either with or without US-guidance, can reduce suffering from lower limb injuries, as compared to current standard of care for pain control in these settings. The study will also evaluate if a brief, focused training for international and local generalist health care providers in RA is sufficient for them to gain competence in the RA procedures.

### Setting

As natural disasters can occur anywhere in the world, the specific geographic location for the RAPID study is not predetermined. Earthquakes occur with greater frequency in Asia and in the Americas, accounting for 55 % and 21 % of events over the preceding four decades, respectively [1]. As such, these are the most likely geographic settings for the trial.

The RAPID study will be restricted to low or middle-income countries (LMICs) as defined by the World Bank classification system [30]. As 70 % of the nations with the largest number of major earthquakes over the past 40 years are classified as LMICs, a resource-constrained setting will enhance the generalizability of the findings to venues with a higher future probability for such events [1]. Additionally, trial activities will take place in an MSF field hospital, which would only be deployed for a major disaster in a LMIC at the request of the local MoH.

**Fig. 2:**
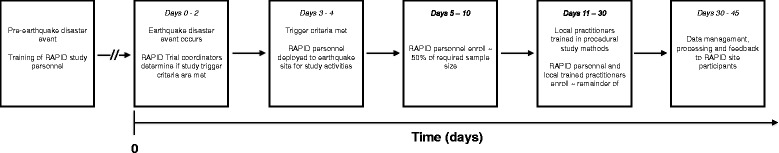
Study timeline

In addition, this trial will only be conducted after an earthquake that meets at least one of the four criteria for a major disaster established by the Center for Research on the Epidemiology of Disasters (CRED): ten or more people reported killed, 100 or more people reported affected, a declaration of a state of emergency, and/or a call for international assistance [31]. The decision as to whether the earthquake meets these criteria will be made within 24 h of the event, to allow for trial commencement within 72–96 h of the event (Fig. 2).

### Outcome measures

**Fig. 3:**
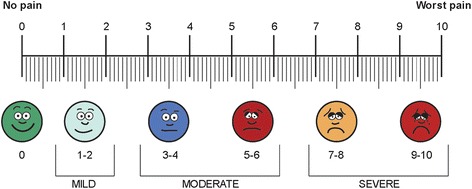
Numerical Rating Scale

The primary outcome measure will be the summed pain intensity difference (SPID), a commonly used measure to assess efficacy of various pain management interventions [25, 32]. While the SPID has not been previously validated in a post-earthquake setting, it has been used in a variety of clinical settings around the world. SPID measures pain over time and controls for both cultural and individual differences in pain perception and expression. To calculate the SPID, participants will be asked to estimate their pain using a standard 11-point Numerical Rating Scale (NRS) at baseline (prior to treatment) and at set time points over the subsequent 24 h of follow-up (Fig. 3). The SPID will be calculated as the area under the curve describing the difference between current and baseline pain intensity at each time point.

Secondary outcome measures will include analgesic requirements, adverse events and patient satisfaction. Analgesic requirements will be recorded as the total amount of analgesic medication received during the full 24 h of patient follow-up. Adverse events (defined as any untoward medical occurrences in subjects receiving the investigational therapy) and serious adverse events (defined as any potentially life-threatening or disabling adverse events) will be monitored and recorded through structured reporting. Patient satisfaction with their overall pain management will be assessed via standardized questionnaires using Likert scales [33, 34].

### Study population

#### Screening

The study population will be comprised of those injured in association with the earthquake who present for care of lower limb injuries at the MSF field hospital. Study personnel will continuously screen all patients presenting to the field hospital until the sample size is reached. Patients meeting all inclusion criteria and no exclusion criteria will be eligible for enrollment.

#### Inclusion/exclusion criteria

The RAPID study will include adult patients (18 years or older) presenting to the MSF field hospital with one or more lower limb injuries. The trial will exclude patients with multisystem trauma, severe respiratory distress, hypotension, altered mental status, active infection at the sight of injection, known current pregnancy, and those who are unable to provide informed consent. Patients with known allergies to local anesthetic agents or narcotic pain medication, those receiving antithrombotic therapy or with a preexisting coagulopathy, and those likely to receive regional anesthesia for alternative reasons within 2 h of screening will also be excluded.

### Informed consent

Study personnel, in conjunction with local translators, will obtain informed consent from all participants prior to enrollment. Since many study subjects may be illiterate, an informed consent script will be utilized that will be read aloud to them and translated into the local language. Subjects will then be able to consent via either a signature or a thumbprint on a Written Consent Form, based on their level of literacy.

### Randomization and blinding

Randomization will occur immediately following informed consent attainment. Study subjects will be individually allocated in a 1:1:1 parallel format into three distinct study arms via blocked randomization with six subjects per block. Allocation will be achieved using sealed envelopes containing computer-generated and preprinted randomization assignments with unique patient identification numbers.

The enrolled participants and the treating health care teams will be blinded to the patient’s allocation arm through uniform exposure to US scanning and injection procedures as outlined below. Only the research team proceduralist delivering the injection will know the intervention received by the patient.

### Allocation arms

The RAPID trial will have three allocation arms (Fig. 1). The first arm will serve as the control group in which participants will receive a standard parenteral injection of 0.1 mg/kg of morphine either intravenously or intramuscularly. Additionally, 5 ml of 0.9 % normal saline (placebo) will be injected into the subcutaneous tissue of the anterior proximal thigh by the proceduralist. Concurrent with the normal saline injection, the US probe will be placed on the patient’s thigh in close proximity to the injection site in order to facilitate blinding.

The second arm will receive the standard 0.1 mg/kg of morphine followed by an injection of 20 ml 0.5 % levobupivacaine into the fascia iliaca compartment. In this arm, the FICB will be performed using standard anatomic landmarks to guide the injection [35]. To achieve blinding during the anesthetic injection, the US probe will also be placed on the skin of the thigh in close proximity to the injection.

The third arm of the RAPID trial will involve treatment with US-guided FNB. In this arm, enrolled participants will receive the standard 0.1 mg/kg of morphine followed by an injection of 20 ml 0.5 % levobupivacaine around their femoral nerve under direct US-guidance [35].

All patients enrolled in the study, regardless of treatment arm, will continue to receive standard care for pain based on the discretion of their treating health care providers in the field hospital. These providers will not be a member of the RAPID research team and will be blinded to the patient’s randomization arm. Enrollment in the study will not be allowed to delay any additional treatments or interventions deemed necessary by the treating team.

### Research personnel training and deployment

Prior to initiation of the trial, and preceding the earthquake event, a cohort of MSF nurses and physicians will be trained in study procedures via a structured interactive course. These practitioners will make up the international portion of the RAPID research team, responsible for data collection and procedural activities. The training will cover all study procedures, including both anatomic landmark- and US-guided femoral nerve blockade for RA [18, 36]. For each trainee, minimum competence will be assessed using a didactic knowledge test and assessment of procedural abilities through direct observation using phantom training models. All research team members will participate in an additional and mandatory Good Clinical Practice (GCP) training.

Within 72–96 h of the earthquake event, members of the RAPID research team will be deployed alongside the MSF response team and field hospital to the site of the disaster. The research team will be divided into shifts, to facilitate 24-h enrollment; each shift will include, at minimum, one data collector, a proceduralist, and a local translator.

As outlined in Fig. 2, study coordinators will also hold an onsite procedural training for locally recruited health care providers, who will take over as the RAPID proceduralists during the latter half of the study. These providers will be trained and assessed onsite using the same methods as the international portion of the research team.

### Data collection

Data collection time points are outlined in Fig. 1. Study personnel will assess the subject’s pain score and vital signs at baseline and again at eight predetermined intervals using the 11-point NRS (Fig. 3). Adverse events will be monitored throughout the follow-up period. Additionally, subject satisfaction with their care and total amount of required analgesic medications will be assessed at the conclusion of the study for each participant. Baseline demographic characteristics and information on mechanism of injuries will also be gathered at the time of enrollment. As the population being evaluated will be followed-up for only 24 h at a single study site and will have substantial physical trauma, it is likely that losses to follow-up through discharge prior to 24 h or patients leaving against medical advice will be minimal.

### Data management

As the trial site will likely be in a setting lacking consistent access to electricity, data will be collected initially on preprinted standardized Data Collection Forms. Forms will be organized based on unique participant identification numbers and contain no personal identifiers. After collection, data will be entered into a password-protected database using REDCap electronic data capture tools by a member of the research team and backed up to an external encrypted hard drive daily [37]. All data will undergo subsequent re-entry after the acute response phase to evaluate for input errors. If a discrepancy between the first and second entry is found, the entry will be reconciled by consulting source documents. The original paper versions of all study documents will be securely stored and accessible only by study investigators.

### Statistical analysis

#### Sample size

The sample size for this study uses a framework of an equivalency trial with parenteral morphine as the standard of care versus morphine with RA as the novel treatment. The new modality is further divided into two arms of RA: FICB and US-guided FNB. For the purpose of this study, a clinically important effect size was defined as a 20 % change in the primary outcome of the SPID. Based on this assumption, a sample size of 63 patients per arm provides 90 % power to show equivalence between each group (e.g. RA administered via FICB as compared to standard care or RA performed using a FNB versus standard care), assuming a standard deviation (SD) up to 20, a difference between the SPID group means of 0 and an equivalence of 13, at a 2.5 % significance level (one-sided). Allowing for 10 % attrition, the sample size is 69 patients per arm, with 207 participants in total.

#### Outcome analysis and reporting

The primary outcome variable, SPID, will be calculated using the pain-intensity difference (PID) at each time point outlined in Fig. 1, which is the difference between the patient’s pain level at that time on a standard 0–10 scale and the patient’s baseline pain level [25]. The SPID will be calculated by summing the pain intensity difference (PID) at each of the study time points, weighted by the amount of time since the prior assessment; this approximates the area under the curve for PID over time. SPID will also be reported as a percentage of maximum possible SPID.

Baseline patient characteristics and outcome measures will be reported using means and standard deviations, medians and ranges, or proportions as appropriate. Analysis of variance (ANOVA) and Student’s *t* test will be employed to determine differences between study arms for continuous measures, including the primary outcome of SPID, and the Fisher’s exact test will be used for differences in categorical variables. Two-way ANOVA testing with repeated measures in one factor (time) will be performed to compare the effect of treatment arm over time on pain-intensity measures.

Missing data will be handled using multiple-imputation models and standard error uncertainty estimates in which missing data will be replaced using summary outcomes from participants with complete data for each variable of interest. To assess the robustness of the models, sensitivity analyses will be performed in which the primary outcome will be compared with and without those lost to follow-up included and assumed to have the mean SPID correlating to the allocation arm of initial randomization.

Prior to trial initiation, a statistical analysis plan with formalized procedural protocols will be created. Analyses will be performed using STATA 13.0 (StataCorp LP, College Station, TX, USA). A *p* value of less than 0.05 will be considered significant for all analyses. All data analyses will be coded on program files to allow independent reproduction of analyses.

Reporting of outcomes will follow the most up-to-date Consolidated Standards of Reporting Trials (CONSORT) guidelines as well as the extensions to equivalence trials; and to nonpharmacological interventions. The collected data set will also be made available in conjunction with the associated trial report.

### Safety monitoring

An independent Data Safety Monitoring Board (DSMB) will review participant safety and efficacy of therapies. The DSMB will be able to recommend modification if there is concern over undue risk stemming from adverse events or other safety concerns. The DSMB will be identified prior to the start of the study and will be activated when the trial commences.

All research methods will be specified in Standard Operating Procedures (SOP). An internal study monitor will be integrated into the research team to assess protocol adherence during data collection. Additionally, the RAPID trial coordinators will be in the field for the study to ensure safe and ethical conduct.

All patients who are enrolled in the trial will be monitored for adverse events. Monitoring will occur via standardized assessments at predefined time points throughout the 24-h follow-up period (Fig. 1). Continuous monitoring by study personnel will be undertaken for a 30-min period following the administration of any study treatment medication to assess for immediate serious adverse events (SAE).

For the purpose of this study, SAE includes hemorrhage, allergic reaction, anaphylaxis, hypotension, respiratory depression, compartment syndrome, intraneural injection, local anesthetic systemic toxicity (LAST), or death. With the occurrence of any SAE, study personnel will initiate immediate treatment in conjunction with the participant’s primary health care team. The onsite coordinator, principle investigators, and DSMB will be made aware within 24 h of any SAE.

### Ethical approval

The RAPID trial has received ethical approval from the MSF Ethical Review Board (Reference number: 1524). The trial will be conducted in accordance with the Declaration of Helsinki, as well as the International Conference on Harmonization (ICH). Collected data will not be linked to any individual or personal identifiers. Confidentiality will be maintained at all levels of data management.

Within 72 h of the earthquake, the research team will attempt to identify an ethics committee in the country in which the disaster occurred, which will be given the opportunity to review the trial protocol and prior ethical approvals and recommend any changes deemed necessary. In the absence of a local ethics committee, the research team will attempt to contact the appropriate department in the local MoH to obtain authorization for the study prior to beginning recruitment at the MSF field hospital. Recruitment will not begin until these local approvals have been obtained. In addition, all attempts will be made to recruit local coinvestigators to support the management of the research study.

## Discussion

Earthquakes cause the greatest burden of injuries among all natural disasters globally [1, 2]. Studies of injury patterns from earthquakes show that approximately half of all patients sustain lower extremity injuries [3–6], and that pain management for these victims is often inadequate [11–13]. The shortage of sufficient narcotic medications in LMIC settings, where the majority of earthquake injuries occur, further compounds this problem [16].

While there are reports on the use of RA for pain control in disaster settings, there have been no controlled studies evaluating whether RA has added benefit over narcotics alone [13, 29]. This trial will rigorously evaluate the role of RA and also be the first to assess if a focused training for generalist health care providers is sufficient to effectively perform RA in the aftermath of a major disaster. Given the novelty of this particular research study, as well as the approach of performing an RCT in the aftermath of a major disaster, the RAPID trial has the potential to transform the management of pain in earthquake settings as well as research in acute disaster settings.

The utility of RA in pain management of lower extremity trauma in high-income country hospitals is well-described [24–26]. However, the injury patterns and clinical parameters of this population are not representative of a post-earthquake setting, where resources are often scarce and injuries more complex. Although there are anecdotal reports on the use of RA from contemporary earthquake events [13, 28, 29], this data is limited and equipoise exists as to whether RA improves pain control for lower extremity injuries of disaster victims. The RAPID study will utilize a blinded RCT equivalence design to address this patient-centered question. Although a nonrandomized observational study would be less resource and methodologically intensive, such a design would likely suffer from confounding as patients with more serious injuries, who are more likely to experience adverse events and less likely to achieve adequate pain control, would have a higher probability of receiving RA. Additionally, as the RAPID study outcomes include assessments of pain, blinding is crucial in order to avoid bias due to the placebo effect and, therefore, necessitates a controlled trial design.

If the RAPID trial demonstrates the effectiveness of RA, the findings will provide high-quality evidence to inform the management of traumatic injuries in disaster settings. Studies conducted after contemporary earthquakes report oligioanalgesia, both for patients with severe injuries and for those undergoing painful procedures [11–13, 28]. RA may help reduce pain and suffering of the large numbers of individuals injured in major earthquakes annually, while also aiding in the provision of more definitive treatments of injuries through fracture and wound management. Beyond earthquakes, the medical literature on conflict-associated injuries also demonstrates high rates of lower limb trauma, which suggests possible application of the RAPID findings beyond earthquake settings to those injuries sustained during other types of humanitarian emergencies [17].

The training of international and local generalist health care providers as proceduralists in the trial will enhance the external validity of the findings, as the likelihood of having access to a large body of specialists trained in RA techniques in a LMIC disaster setting is low. In addition, RA can be performed with local anesthetics that have a nominal cost per dose to administer, making it a very cost-effective intervention in even the most resource-limited settings. If the hypothesis of this trial is proven, the use of regional anesthesia for painful injuries after disasters may serve in subsequent disasters to be both a very cost-effective and locally appropriate method to improve pain management and reduce long-term morbidity and post-injury disability [12, 17, 38, 39].

To date, there have been no studies anywhere in the world evaluating the effectiveness, safety, or acceptability of RA in the aftermath of a major earthquake. Indeed, there have been no RCTs evaluating any acute patient-centered medical intervention in the aftermath of a major earthquake. Therefore, beyond addressing the immediate need for improved pain management in these settings, the RAPID study is also pioneering new methodologies in humanitarian and disaster research. Although the evidence base for humanitarian response is replete with retrospective studies, there is a dearth of high-level evidence from prospective trials to inform the care of disaster victims [40]. This deficit results in the use of data extrapolated from nondisaster populations and consensus opinions for developing treatment recommendations in disaster settings [14]. As such, execution of a rigorous RCT in an acute disaster setting will serve to improve the current evidence base, future research activities, and subsequent humanitarian response guidelines.

With the high burden of disease, consistent patterns of injuries and inadequate pain management documented in earthquake settings, new interventions to address this substantial global health problem are needed. The RAPID study will be the first prospective RCT to evaluate whether RA administered by generalist health care providers, either with or without US-guidance, can safely and effectively reduce suffering and pain from earthquake-related lower limb injuries in a disaster setting.

### Trial status

Ethical approval for the study has been obtained from the MSF Ethical Review Board and 12 study personnel from MSF have been trained in the trial SOP. The study will commence after a major earthquake meeting the criteria outlined above occurs in a LMIC to which MSF has been asked by local government to deploy a field hospital.

## Abbreviations

CRED: Center for Research on the Epidemiology of Disasters
DSMB: Data Safety Monitoring Board
GCP: Good Clinical Practices
ICH: International Conference on Harmonization
LAST: Local Anesthetic Systemic Toxicity
LMIC: Low- and middle-income country
MoH: Ministry of Health
MSF: Médecins Sans Frontières
NRS: Numerical Rating Scale
PID: Pain intensity difference
RA: Regional anesthesia
RCT: Randomized controlled trial
SAE: Serious adverse events
SPID: Summed pain intensity difference
US: Ultrasound
WHO: World Health Organization
